# Context counts: a qualitative study on how adolescents activate social resources to develop and practice health literacy

**DOI:** 10.1186/s12889-024-21138-9

**Published:** 2024-12-24

**Authors:** Rebekah A. Hoeks, Michael J. Deml, Olivier Favre, Oliver Senn, Saskia Maria De Gani, Yael Rachamin

**Affiliations:** 1 Institute of Social Anthropology, University of Basel, Münsterplatz 19, Basel, 4051 Switzerland; 2 Institute of Sociological Research, University of Geneva, Geneva, Switzerland; 3 Department of Child and Adolescent Health, Office of Public Health, Canton of Zug, Zug, Switzerland; 4 Institute of Primary Care, University of Zurich and University Hospital Zurich, Zurich, Switzerland; 5 Careum Center for Health Literacy, Careum Foundation, Zurich, Switzerland; 6 Careum School of Health, Kalaidos University of Applied Sciences, Zurich, Switzerland

**Keywords:** Health literacy, Social resources, School health services, Qualitative research, Adolescence, Switzerland

## Abstract

**Background:**

Recently, the importance of social networks and other contextual factors in shaping health literacy of adolescents has gained recognition. However, research often simply refers to *context* without explicitly describing it. In this qualitative study, we aimed to explore how adolescents activate their (social) resources to develop and practice health literacy within a Swiss cantonal school health service program and in their everyday lives.

**Methods:**

This study is based on a secondary analysis of interviews from an evaluation of the school health service in the canton of Zug, focusing on the final health screening in the 7th grade (corresponding to an average age of 14 years). Semi-structured interviews were conducted with 16 students. The data was analyzed using thematic analysis and constructivist grounded theory to identify and refine key themes.

**Results:**

Our analysis revealed that adolescents’ interactions with their social networks, including family members, teachers, healthcare providers, and peers, were significant resources in their health literacy practices. Other resources included school health services, regular curricula and the internet. The activation of these resources was moderated by several factors that functioned as *activators* or *deactivators*, which we divided into three categories: *relationships*, *health system*, and *pre-existing health literacy*. Prominent activators in the category relationships involved *good rapport* and *trust*, whereas *fear of judgment* deactivated resources. In the category health system, *access* to resources as well as opportunities for *participation* in dealing with health information, challenges and services were important activators (or when missing, deactivators). Finally, participants demonstrated that pre-existing health literacy in the form of *pre-existing knowledge* and *motivation and attitudes* served as an activator of their resources to develop and practice health literacy in a ‘virtuous circle’. Thus, although health literacy development and practice were dependent upon their social networks, adolescents played key roles as active agents while navigating health.

**Conclusions:**

Our findings highlight potential (de)activators of adolescents’ resources, primarily those available within their social networks, in the development and practice of health literacy. Results contribute to the literature on adolescent health literacy by shedding light on the often under-described concept of context. Explicit consideration of context provides actionable insights for educators, healthcare providers, and policymakers seeking to support adolescents in the development and practice of health literacy.

**Supplementary Information:**

The online version contains supplementary material available at 10.1186/s12889-024-21138-9.

## Background

### Introduction

In the past two decades, the concept of health literacy has been established as an important driver of health, with low health literacy being linked to various negative health outcomes [1]. Broadly defined as “a bundle of competences to proactively deal with health-related information, services, and challenges and, thereby, [empowering] people to manage their and other’s health and well-being” [2, p. 14], health literacy encompasses a wide range of knowledge, motivation and competencies essential for making informed decisions about health [3]. In recent years and particularly with respect to children and adolescents, health literacy has gained recognition as being a dynamic construct shaped not only by individual competences, but being highly *relational* and influenced by *context* [2, 4–6]. However, the scope of said context often remains ambiguous.

Health literacy is particularly important in adolescence, as it not only affects immediate health behaviors but also lays the foundation for lifelong health practices before adolescents’ transition into adulthood. One way to strengthen adolescents’ health literacy is through school health services [7, 8], as there is a history of promoting health literacy in educational settings and programs [9, 10]. However, campaigns during the 1960s and 1970s in the Global North were mainly concerned with transmitting health information without considering the social and economic circumstances of their audience. A significant shift took place in the 1980s, when the complex connections between knowledge, beliefs, social circumstances and norms were taken into account. Such later campaigns often led to step-by-step implementation into school-based programs [10].

A similar shift has taken place in recent years in school health services in Switzerland, with cantons focusing more on health promotion, prevention and health literacy [11]. In Switzerland, school health services – similarly to healthcare and education – are federally regulated and organized at the cantonal (regional) level [12]. In most German-speaking cantons, the cantonal or municipal administration assigns the school health service with the task of conducting school medical examinations, with screenings occurring several times during compulsory school. In the past couple of years, several cantons, including Zug, have introduced new elements to school health services with the aim of increasing students’ health literacy. Nonetheless, the implementation of health literacy programs in schools remains limited.

### Literature review

The term health literacy was first introduced in the 1970s [9]. In subsequent decades, health literacy efforts focused heavily on individuals’ reading and numeracy skills in order to function in healthcare settings [13]. Nutbeam [10] expanded the definition of health literacy beyond functional skills to include interactive, communicative and critical skills to understand, communicate and act on health information. Since then, the concept has further evolved [14], e.g. including media literacy as a fourth type of skill [15]. Generally, a more holistic view was adopted [16], which was further developed by Sørensen et al. [3, p. 3], culminating in their widely referenced definition: “Health literacy is linked to literacy and entails people’s knowledge, motivation and competences to access, understand, appraise, and apply health information in order to make judgments and take decisions in everyday life concerning healthcare, disease prevention and health promotion to maintain or improve quality of life during the life course.” We have informed this article with the definition of De Gani et al. [2], who go a step further by including competences not only to proactively deal with health-related information, but also to navigate health services and challenges.

As previously mentioned, recent literature on health literacy has often emphasized the importance of an individual’s context when making health decisions, in particular for children and adolescents, but does not often clearly delineate what is meant by this beyond the surface level [4, 6]. Health literacy research could benefit from a clearer understanding of context because it is often in contextual elements where opportunities for change can be identified. That said, some models have made efforts to describe context; for instance, Wharf Higgins et al. [5], propose a social-ecological model of adolescent health literacy, which highlights three different levels that influence an individual’s health literacy: intrapersonal factors (characteristics, knowledge and skills), interpersonal factors (social support, quality of human interactions, peers, family etc.), and community (environmental and structural) factors. In addition, Bröder and Carvalho [17, p. 40] have argued that “research should address how children and adolescents actually mobilize their resources and capabilities to practice healthy decisions in the context of their everyday life.”

Some researchers have made attempts to go beyond skills and deficit model-based understandings of health literacy. For example, Papen [18] challenged the dominant view of health literacy as abstract attributes and generic abilities, or skills, and rather argued that it is a situated social practice, and that health literacy can be “distributed” among individuals and is embedded in social relationships. She focuses on health literacy from a resources-perspective rather than a deficit-perspective. Edwards et al. [19, p. 1190] further developed distributed health literacy as “the health literacy abilities, skills and practices of others that contribute to an individual’s level of health literacy.” Others have emphasized the importance of emotional support from the social network for an individual’s health literacy development and practice, arguing that people are not only looking for facts to solve their health concerns, but are actually also seeking social support from friends and relatives for comfort and orientation [20]. Building on previous discussions of context, Pithara [6] proposed to re-think health literacy through a capabilities approach lens, arguing that the focus should be on health literacy capabilities and enabling or inhibiting factors shaping health literacy instead of individual skills and competencies. Recently, De Gani et al. [2, p. 14] introduced the concept of “health literacy enablers” as “practices, processes, structures, and policies of various actors within and beyond institutional, sectoral, or regional boundaries through which people are empowered to develop and strengthen their health literacy” to account for contextual factors that (positively) influence the development and strengthening of people’s health literacy.

### Research question(s)

In 2019, authors YR and OS were approached by the canton of Zug to independently evaluate its school health service. In 2015, Zug had introduced a reorganization of these services, which aimed to better integrate health promotion and prevention topics, and to increase students’ health literacy [21]. The evaluation thus focused on these new elements and their effectiveness. A mixed-method study was conducted with 7th grade students (i.e. 14-year-olds), which included semi-structured interviews with volunteer participants. In the process, participants provided important insights into their health literacy development and practices which went beyond the scope of the school health service evaluation. Subsequently, a team of experts in medicine, health services research, health literacy, sociology and anthropology was gathered to qualitatively analyze these interviews and delve further into the topic of health literacy among adolescents to explore how they develop and practice health literacy in the context of a Swiss cantonal school health service program and in their everyday lives.

Given that distributed health literacy has been predominantly researched in children, youth and minority groups [22], and the emphasis on the importance of an individual’s context particularly for children (and other vulnerable groups) [4], we were not surprised to find that participants’ social networks and broader structural, relational and personal contexts played a significant role. Through our analysis, we aimed to shed light on the often ambiguously described context(s) impacting adolescents’ development and practice of health literacy. Thus, through several rounds of iterative analysis and copious literature consultations, we sought to answer the following question: *“What are the positive and negative factors that influence the activation of adolescents’ resources and social networks to develop and practice health literacy and how do they do so?”* Throughout this paper, the practice and development of health literacy are considered as inherently linked, since both form each other; practice is a process of development, and development is fueled by practice.

## Methods

### Setting

The study is based on the secondary analysis of interviews conducted in the canton of Zug in 2020–2021 as part of an evaluation of the school health service. In the canton of Zug, the school health service encompasses three screening examinations during compulsory school, which are conducted by pediatricians or general practitioners (with support of their practice staff) on a part-time basis. In the last examination in the 7th grade (student average age: 14 years), examinations include measurements of weight and height, as well as assessments of hearing, vision, and the back for scoliosis, and a counseling consultation with the school doctor. Moreover, a preparation lesson (prior to the school doctor consultation) is conducted by prevention specialists of the canton of Zug who visit the schools. In the preparation lesson, students receive information about health topics, when and where to seek help in case of health-related challenges, and what will happen during the school doctor examination. Additionally, all students complete a self-assessment questionnaire to evaluate their health status and behaviors related to various health topics like well-being, substance use, and bullying. An evaluation of this questionnaire is then brought along to the school doctor appointment to serve as the basis for their discussion [23]. This process was designed to encourage students to reflect on their health status and behaviors, to serve as a reminder of any health concerns they wish to address with school doctors, and to enable school doctors to pinpoint the health issues that are of the highest priority to the students [21].

### Data collection and participants

Qualitative data were collected via semi-structured interviews. To recruit students for participation in the interviews, informational leaflets were distributed in the preparation lesson with contact information of the study team and, alternatively, a QR code through which students could easily register for the interview. Moreover, students could declare their interest in participating in the interview in a digital survey they filled out as part of the school health service evaluation at school. The study team subsequently contacted interested students to schedule a meeting. For participation, signed consent from parents was required, and students received 20 CHF compensation. In total, 16 students were recruited and participated in the study. The participants (75% female) came from 12 different school classes and 8 different schools (of 14 total) in the canton of Zug. They were all in 7th grade. Two participants were in educational track level C (lowest educational level), 8 in level B, and 6 in level A (highest educational level). The interviews were meant to be held in person at the *Amt für Gesundheit* in Zug, but the COVID-19 pandemic forced a switch to online interviews after four interviews. In the interviews, students were asked about their perspectives on the preparation lesson (including the self-assessment questionnaire) and the school doctor visit, in particular the conversation with the school doctor. The interview guide, which was developed for this project, was then updated based on the results of the online survey and the responses in the interview [see Additional file 1]. The interviews lasted 12 min on average (range: 8–18 min). Pseudonyms are used in this article to protect participants’ privacy.

### Data analysis

The data analysis for this study employed a combination of thematic analysis and grounded theory methodologies to ensure a comprehensive examination of the qualitative data collected from the interviews. The analytical approach first involved use of deductive thematic analysis [24], by which we organized our data around elements of health literacy as defined by Sørensen et al. [3], including but not limited to knowledge, competences and motivation to understand, access, appraise, and judge health information. Using these predetermined categories provided a structure for indexing the data, ensuring that the analysis remained focused on the key themes identified. This allowed us to make initial categorizations, but we quickly realized that this coding scheme was insufficient and too narrow for analyzing our data due to its focus on health literacy per se rather than the context shaping this health literacy.

Subsequently, constructivist grounded theory techniques, as described by Charmaz [25], were utilized to further refine the themes and develop a more nuanced understanding of the participants’ experiences which often centered around their social networks in complex and diverse ways. This approach allowed for the emergence of new categories and subcategories directly from the data, ensuring that the analysis remained grounded in the participants’ perspectives. Constant comparative methods were used to compare data across interviews, which helped in refining the themes and ensuring the credibility and trustworthiness of the findings. Throughout the analysis, the principles of reflexivity and researcher triangulation were maintained to enhance the validity of the results [26]. This entailed authors RH and YR independently coding the data and then discussing their interpretations, consistently and iteratively going back to the data to reach a consensus on the emerging analytical categories and interpretations. This was followed by rich discussions within the multidisciplinary team. Our collaborative approach led to minimized bias and ensured a more robust and reliable analysis.

## Results

“Well, my brother goes to the pediatrician very regularly because of his illness, and if I have a question, I ask my mother. If she doesn’t know, she asks the pediatrician, and then gives me feedback. Otherwise, I just google on the internet and then just collect different sites and put it all together. Or I ask my teacher because my teacher is also very interested in the subject of health. I always have access to him, and I always have had a relatively good relationship with my biology teacher.” (Anna, f, track B).

When responding to a question about how she obtained health-related information, this participant’s response saliently exemplifies how study participants described being embedded in social networks where certain available resources could potentially be used to develop and practice health literacy. We see how Anna navigated health-related information emanating from her social networks, particularly from her brother’s pediatrician, her mother, and her biology teacher. She was also able to gather information found on Google searches and websites on the internet. The activation of these resources was moderated by several factors: Her *rapport*, *trust and familiarity* allowed her to effectively engage with information available from her mother, pediatrician, and biology teacher. She alluded to her *access* to the health system in recognizing the availability of the pediatrician as an arbiter of health expertise. Furthermore, she made use of her *pre-existing health literacy* by showing her *knowledge* and *motivation* in combining internet sources.

**Fig. 1:**
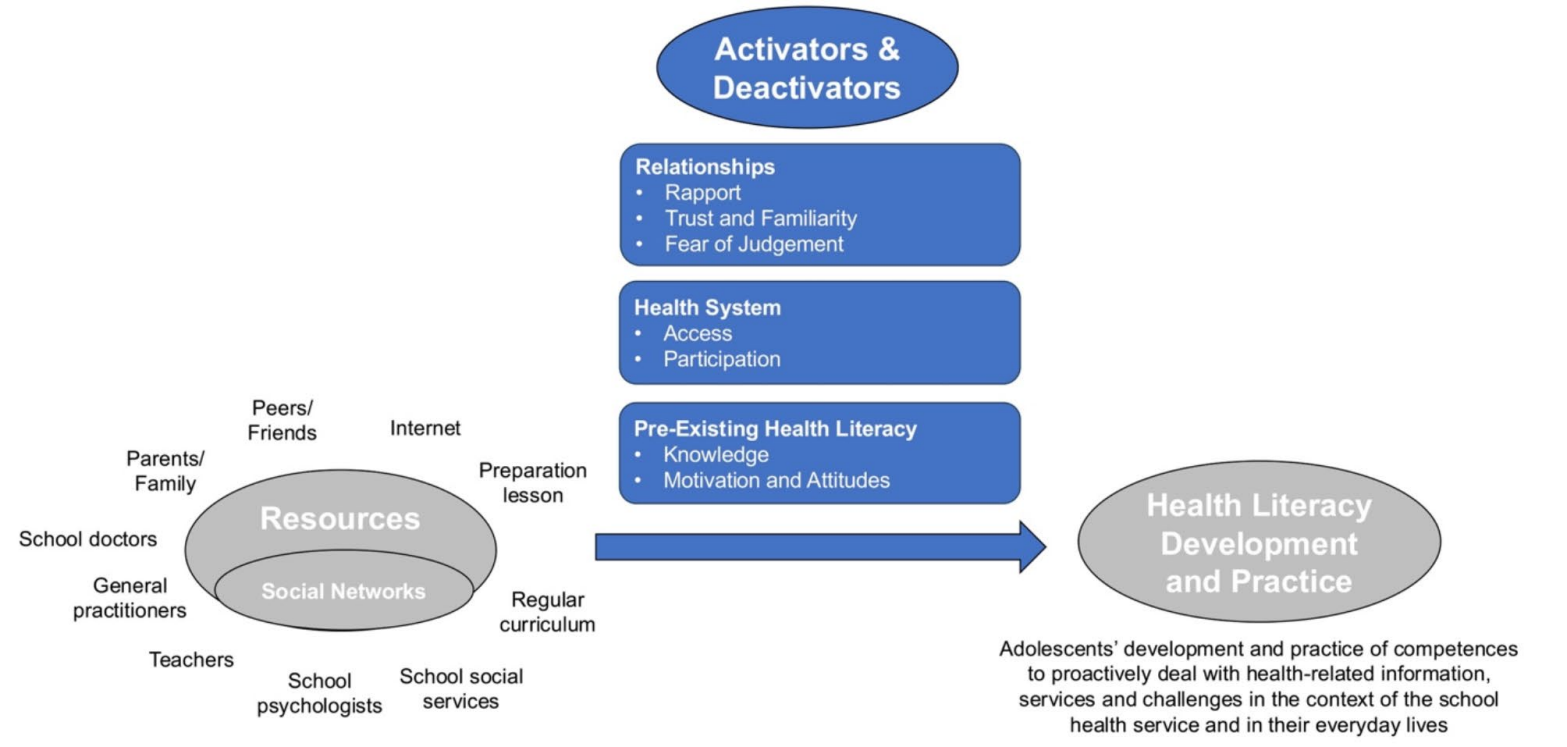
Graphic representation of activators and deactivators which moderated the activation of adolescents’ resources to develop and practice health literacy

Analysis of our interviews showed how health literacy development and practices were moderated through the processes of activation or deactivation of resources available to participants via their social networks. As will be demonstrated in the following sections, whether or not participants were able to harness the resources available to them was contingent upon an array of contextual factors, which we refer to as *activators* and *deactivators*. It is also important to note that adolescents were not necessarily aware that resources were being activated or deactivated; the activators and deactivators were identified during data analysis. To account for the complexity of how adolescents interacted with their social networks and the resources available to them, we introduce a graphic representation of the processes at play as adolescents strive to deal with health-related information, health challenges and health services (see Fig. 1). In the following sections, we further detail how our data informed the identification of different activators and deactivators in the following categories: (1) *relationships* (*rapport*, *trust and familiarity*, *fear of judgment*), (2) *health system* (*access*, *participation*), and (3) *pre-existing health literacy* (*knowledge*, *motivation and attitudes*).

### Relationships

It became clear in our interviews that the *relationships* participants had with their social surroundings had a very high potential to (de)activate these resources. The duration and quality of relationships that adolescents had with their peers, parents, health professionals and teachers varied to a great degree and were dependent on different circumstances. Nonetheless, when a certain quality of relationship was not met, participants were less likely to activate their social network in order to deal with health challenges, health services or health-related information. Often, relationships were connected explicitly to disclosure of health information, ranging from direct health concerns to general insecurities. In the following sub-sections, we describe the various levels of relational dynamics participants commonly expressed in the context of their health: *rapport*, *trust and familiarity*, and *fear of judgment*. We describe how these considerations influenced the activation of their social networks as resources for developing and practicing health literacy.

#### Rapport

Our interviews highlighted that a good *rapport* was an important activator of resources for participants, in particular in healthcare settings. Many participants mentioned having a good rapport with healthcare professionals in their area, as for example Clara (f, track B): “Here in [our village], our doctors are very good and cool and so I personally wasn’t afraid and neither were the others.” Some students mentioned the school doctor’s qualities: “He was very nice, actually, and he also had a great sense of humor and was actually quite sweet” (Sabine, f, track A). Rapport was also linked to having a frame of reference or any kind of relation to health professionals, as Emma (f, track B) for example answered in response to whether she would consult the school social worker: “Well, I don’t know, maybe I would. (.) But I have no reference at all [Pause], so I didn’t even know who it was until she came by. I wouldn’t recognize her either [Pause] and I forgot that she existed.” This participant expressed having no reference to the school social worker, which completely deactivated her as a resource. Research has found that for adolescents, being given a frame of reference helps them understand certain structures, after which they can build a personal relationship with certain health professionals, and knowing how to communicate within such a system is helpful [27]. Such a frame of reference could also act as a deactivator, however. This occurred when health professionals were perceived negatively.

While having a good rapport with healthcare professionals is a key facilitator in accessing healthcare services, feelings of being dismissed or a lack of genuine concern are seen as barriers [28]. Anna (f, track B) described being brushed off quite rudely by the assistant of the school doctor: “Then I went to the eye test, and I have glasses, then it was just quite strange and I didn’t quite know what to do, when she first said, ‘leave your glasses on’, and then she said, ‘I told you to take your glasses off!’ And yes, it was a bit strange, um, not rude but they were very reserved.” She went on to describe feeling dismissed quickly in her school doctor consultation: “Then [the school doctor] looked at my questionnaire and said, ‘Yes, that looks very good, um, bye.’ And I was like, ‘Okay, then the questionnaire won’t be looked at more closely, will it?’ Yeah… very peculiar.” She expressed confusion because she thought that they would talk about the results of the questionnaire she filled out in the preparation lesson, but they did not. A more detailed focus on (de)activators of the social resource of the healthcare provider will be elaborated further in the ‘Participation’ sub-section.

#### Trust and familiarity

*Trust and familiarity* played a crucial role in helping students to activate resources within their social network. Our interviews revealed that students found it easier to disclose (intimate) health information and seek advice when they trusted their social environment. Trust was often tied to confidentiality and familiarity, the latter supporting Rotenberg et al. [29], who argued that familiarity underpins trust. Conversely, a lack of trust often led to a deactivation of participants’ social network as resources. Familiarity, however, both functioned as activator and deactivator, depending on the situation. Below, we provide examples of how trust and familiarity operated as (de)activators for adolescents when engaging with different resources, namely healthcare professionals, teachers, parents, and peers, to develop and practice health literacy.

Many participants described a growing familiarity and trust with their general practitioners (GPs) over time. In some cases, participants were familiar with their healthcare professionals through their parents. Whereas this increased familiarity and could therefore facilitate activation of this resource, there may be a deactivating element related to trust, as research has found that adolescents would like to have a GP who was not their family physician [27]. Emma (f, track B) described the nuances of trust and disclosure in this relationship as follows: “I used to think [the doctor] was a bit weird because he always joked around with my mother when she came too, but he’s just such a nice guy and I know that he wouldn’t tell my parents [anything]. It’s good that he already knows me and he’s known me since I was very little, and I think he would talk to me. If he had a feeling he would say to me, ‘Maybe it would be good if you discussed it with your parents’ or something. But I don’t have the feeling at all that he would tell my parents.” Milena (f, track B) had a similar opinion, stating that her GP would not tell her parents anything that she explicitly asked her not to. Interestingly, Hannah (f, track B) found that the lack of familiarity with the school doctor facilitated disclosure: “I felt more comfortable sharing sensitive information with the school doctor because I might not see her anymore as she’s not my GP and then you can kind of tell this person just once, so to speak.” This anonymity could help in overcoming *fear of judgment* (elaborated on in the corresponding sub-section below), as Sofia (f, track A) explained that not knowing the school doctor personally made it easier to confide in him without fear of judgment or the danger of damaging friendships.

Beyond healthcare providers, and consistent with other literature [30], the interviewed adolescents discussed the importance of non-peer confidants when it came to navigating their health. Such confidants included teachers and parents, for example. Participants had mixed feelings about discussing health topics with teachers, depending on their relationship and trust level. Parents were also often mentioned as resources for advice or just lending a listening ear. For example, Emma (f, track B) explained, “I think if I had a problem that didn’t concern my family, I would first discuss it with my mother, but yeah, I have a pretty good relationship with her.” This indicated that while their good relationship made her mother a resource for medical problems, it also introduced hesitancy regarding family issues, something other participants also mentioned. Flexibility in seeking advice from different resources for different problems was common among participants.

Peers also played a significant role as resources, with trust and familiarity influencing the willingness to share health information. When discussing her self-assessment questionnaire, Laura (f, track B) put it this way: “So I think for me it was ok. Because my friend was next to me and I can tell her everything anyway. But she did have a good look at my sheet. And yeah, if it had been someone else, then that might have sucked a bit.” It mattered to Laura who could see the information she provided in her questionnaire and how trustworthy they were. This was something other participants mentioned, too. When peers were not trusted, resources such as the preparation lesson and school doctor visit were deactivated. Emma (f, track B) described such an instance where the school doctor consultation did not take place in a private, individual setting. She did not want one other peer specifically to find out about anything she could have discussed with the school doctor. She went on to explain how classmates would disseminate information quite rapidly, even if unintentionally, something important to note in consultations that were not in private: “It goes around so quickly, I’m not saying intentionally […] you say it in confidence, but then word gets around anyway.” Here, a lack of trust was related to the fear of judgment by others, which is elaborated in the next sub-section.

#### Fear of judgment

One of the main deactivators of potential resources was *fear of judgment*, which was often a significant barrier to trust. During our interviews, we noticed that there was an atmosphere of judgment and evaluation between adolescents. This was reflected not only in how participants were concerned about being judged by others but also in how often they referred to and judged their peers. This was coupled with participants mentioning situations that were “unangenehm,” meaning “uncomfortable” or “awkward”, which in some cases was related to shame. For example, when asked about who he would turn to with health-related problems, Timo (m, track B) said: “I find it really uncomfortable/awkward to talk about that with other people.” From our data, we found that participants were more likely to express fear of judgment among peers than among adults.

Many participants expressed a fear of their peers and classmates finding out about more private health information. Similarly, students alluded to the importance of having some privacy when filling out the questionnaire on their health status and behaviors: “We were told that everyone had to fill in [the questionnaire] individually, which I thought was good, having a bit of privacy. Because then you weren’t under any pressure to tick a certain box just so others don’t think badly of you” (Emma, f, track B). Hannah (f, track B) mentioned concerns about disclosing certain information to her current friends because their friendship might not last into the future. She explained, “Sometimes, I’m afraid that if I say something to my friends, it can always happen that you don’t get on well with them later, and then word gets out at some point.” However, somewhat in contrast, Milena (f, track B) mentioned that she would entrust peers with more “uncomfortable” information than adults. Such “uncomfortable” topics may be related to mental health, sex, and drugs. This is similar to the findings of Olsson et al. [31, p. 223] in a study with Swedish adolescents showing that the support from parents was preferred for topics related to “home and school”, whereas peer support was preferred for “low mood” and “sex and alcohol.” Wharf Higgins et al. [5] similarly found that adolescents entrust peers more with intimate information.

Not only was the fear of judgment a vital factor for participants when it came to disclosing health information, Hannah (f, track B) actually expressed the importance of disclosing certain information as justification to not be judged: “For example, one [student] once went [to the school social worker] because of her grades (…) [and] she told us about it and it was okay for us, and then that was that. But I have the feeling that if a happier person who doesn’t let anything show – it’s a bit strange when you see that they’re with the school social worker.” She emphasized that this classmate’s explanation for visiting the social worker led to overall acceptance from the class. If her classmate had not done so, or if she had seemed like a “happy person” and then gone to the social worker, everyone would have been asking questions, talking about it and possibly judging the situation. This example shows a type of peer evaluation of judging whether someone’s visit to the doctor or other health personnel is acceptable or not. Participants often referred to and judged others during our interviews. We argue that because they were so concerned with other people, this in turn highlights the importance they placed on the opinion of others.

### Health system

Beyond relationships, *access* and *participation* also played crucial roles in participants’ resource activation. In this section, we focus on certain structural health system factors that are largely outside of the adolescents’ control but influence their ability to activate their resources. By *access*, we refer to the extent to which adolescents were granted access to potential resources to develop and practice health literacy. When discussing *participation*, we describe how healthcare professionals and the preparation lessons engaged adolescents in health discussions and decision-making processes.

#### Access

*Access* to resources such as information, health services, and the social network was crucial for adolescents to be able to activate them. In fact, access may even be viewed as a prerequisite rather than merely an activator. Participants mentioned several resources they could access in case they had a question or problem and needed to talk, including the school social service, their parents, doctors, and teachers, for example. Importantly, a prerequisite for students to access their resources was knowledge of these resources, which is discussed below in the ‘Pre-existing health literacy’. We found that access to participants’ resources was sometimes conditional, e.g. depending on the situation. Timo (m, track B) for example mentioned that he had access to a GP (as opposed to the school doctor) only when he had a specific problem, not just “to talk”: “No, so they don’t invite me, I only ever go there when I have a problem. For example, I have a pollen allergy and then we just talked about pollen. But not about the other topics or anything like that.” Moreover, Timo mentioned that the consultation with the school doctor was the only opportunity he had had to ask questions about sexual health, because the school social service was “for different kinds of problems,” and at school, the topic was only covered very briefly.

Participants often mentioned having enough time as an activator – or rather, not enough time as a deactivator – of their resources. This illustrated how access was not simply black or white. Instead, it existed along a spectrum, with more time being more beneficial. Ria (f, track B) reasoned that the restricted time of a ten-minute consultation may have been a barrier to talking to the school doctor about problems. Time was also mentioned by participants as being an important activator of thinking about their own health, or specifically, activating the self-assessment questionnaire as a resource in the preparation lesson. Hannah (f, track B) for example mentioned that it was good to have some time alone to think about these topics. When asked during the interview if she had not done this previously, she pointed out how there was usually no room for that because of the other things on her mind: “I would say that when I’m at home, I don’t think about things like that so much, I think about homework and what I still have to do and sports training and things like that.”

#### Participation

Keij et al. [32] posit that health literacy is an important element needed for patients to be ready for shared decision-making, or *participation*. We found that in turn, participation could act as an activator (or lack thereof as a deactivator) of resources for the development and practice of health literacy. In effect, we noted the importance for adolescents of being offered opportunities to participate in health decisions and discussions in ways that made sense to them. This participation could either be facilitated or hindered by actors and institutions in the health system, such as healthcare professionals or school health programs.

A participative environment is dependent on feelings of being respected and not being talked down to. For healthcare professionals, this often means using simpler language [20, 33]. As Anna (f, track B) mentioned: “[The school doctor] talked quite quickly and used foreign words that I didn’t understand. Then I asked what that meant and then she explained it again with other foreign words, and that was quite odd. Yes. I remember it was very weird [laughs].” The use of jargon thus deactivated the school doctor as an effective resource. Many participants also mentioned the opportunity to ask questions, and being asked questions, as an activator in the school doctor consultation – or the lack thereof as a deactivator. Laura (f, track B) nicely demonstrated how being asked a question does not necessarily increase participation: “Yes, so [the school doctor] sometimes briefly said, ‘Do you have any questions?’ and then he just carried on.” Many students expressed not having any questions for the school doctor, but wished to talk more nonetheless. This could be indicative of the school doctor needing to ask direct questions, fostering active listening and a participative and inviting environment, rather than expecting this from the adolescent.

A recurring theme was the wish for more adequate or more detailed information during the school doctor consultation, indicative of the current situation hindering participation and thus activation of the school health service. Reto (m, track C) for example expressed: “[The school doctor] took my blood pressure, and I thought that was very fast, so he just pressed a button and then just took it out again. [.] I couldn’t even read what it was.” Raymundo et al. [28] also found that a certain level of detail is welcomed by adolescents during their healthcare consultations. Similarly, it did not make sense to our participants that they were measured and weighed with shoes and clothes on, but this was neither addressed nor explained by the health personnel.

Participation was also an important activator in the preparation lesson, as for example Sofia (f, track A) mentioned: “I particularly liked the fact that he asked us a bit first and looked at what we already knew and then added a bit more, because that allowed him to adapt a bit to us.” Hannah (f, track B) also mentioned how having a safe space to make mistakes enabled the class to participate and activate the resource of the preparation lesson. In contrast, Anna (f, track B) and her class’s participation lesson was more of a lecture and only a short amount of time was spent talking about what would actually happen during the school doctor’s visit: “So we talked about health for 40 minutes and then the last five minutes, well, it wasn’t even five minutes, it was a minute, if anything…Then she said: ‘Ah, this and that is happening, goodbye everyone’. It was just very strange, you couldn’t ask questions.” Here, participation was not encouraged, deactivating the resource of the preparation lesson.

### Pre-existing health literacy

The final set of factors we identified that moderated the activation of resources to develop and practice health literacy was the participants’ *pre-existing health literacy*. In the following sections, we describe *pre-existing knowledge* as well as *motivation and attitudes* to deal with health information, health services, and health challenges, that led to participants (de)activating their social networks to further develop their health literacy.

#### Pre-existing knowledge

During interviews, participants demonstrated many instances of *pre-existing knowledge* regarding health information, health services, or health challenges, and demonstrated how such knowledge enabled them to activate their resources to develop and practice health literacy. For example, many participants demonstrated health literacy in the sense of knowing who to turn to for health information or support and critically evaluating which resource they would activate in specific situations. The ‘virtuous circle’ of pre-existing knowledge is also nicely exemplified by the preparation lesson and the school doctor consultation. Through activation of the preparation lesson as a resource, participants were prepared for what they wanted to talk about with the school doctor and increased their understanding of the school health service, in particular, what they could expect in the consultation, which in turn enabled them to make better use of the school doctor as a resource. In contrast, a suboptimal understanding of the aim of the school doctor consultation led to participants not effectively activating this resource. Milena (f, track B) for example took the doctor’s word literally when invited to ask questions about a topic she was least informed about, thinking she could not ask questions about a topic she was actually interested in but already had some knowledge on. This is in line with previous research showing that “not knowing how to ask the doctor the questions I really wanted answers to” was among the top three doctor-patient communication problems of high school students [27, p. 190].

Participants demonstrated varying degrees of critical thinking in regard to the evaluation of the self-assessment questionnaire they filled out. Clara (f, track B) for example shared her experience of how some results of the questionnaire were based on past information which was not indicative of her current health status. This led to a misunderstanding, which she was able to resolve by communicating with the school doctor: “I think the questionnaire should be a bit more precise because, for example, it says ‘Have you ever been bullied?’ and I think a lot of people, well, I think everyone has been bullied at least once in our class. But the thing is, if you say ‘yes’, the result [of the questionnaire] is that you’ve been bullied a lot, but it might have been in 5th or 6th grade. And then the doctor is like ‘Ah you’re being bullied’ and I’m like ‘No! Not anymore.’” This illustrates how her advanced understanding of what the questionnaire was meant to reflect – indicating high health literacy – was crucial to navigating the school doctor consultation. Participants also demonstrated maturity and critical thinking in stating that the questionnaire was only beneficial if they answered honestly. Knowing this encouraged Timo (m, track B) to be honest, leading to a better understanding of his health status: “If you weren’t honest with yourself, you didn’t get the right answers, but I was honest. And now I’ve actually discovered what’s really important to me.”

#### Motivation and attitudes

Participants’ *motivation and attitudes* also acted as (de)activators of their resources. For instance, participants shared their attitudes on health and the role of healthcare providers, and in line with Bhagat et al. [34], participants’ conceptualizations of health and health personnel seemed to have had an impact on the way in which they activated these resources. When asked whether she or her peers would talk to the school doctor about their problems, Laura (f, track B) answered: “I feel like probably not. I think he’s a doctor, somehow, not a psychologist. Personally, I haven’t spoken to him about my problems.” Believing that a physician was only for physical problems was mentioned by several participants and acted as a deactivator of this resource, consistent with results of Eigenhuis et al. [35], who found that this belief was one of the reasons mentioned for adolescents and young adults not going to the GP with depressive symptoms.

Participants also talked about their motivation to learn about health topics. We argue that pre-existing motivation to access, find, and generally deal with health-related information and challenges enabled participants to develop a higher level of health literacy. For example, one participant mentioned career aspirations in the health sector, which had previously led her to activate her resources to gather knowledge on health topics. Additionally, participants demonstrated motivation to deal with health information and challenges to improve other people’s health, as illustrated in Emma’s (f, track B) statement on substance (ab)use: “I think it would be good if this was brought up and people didn’t just look past it.” In part, this seemed to be related to the fact that the health behaviors of others impacted participants’ own health. Emma later went on to describe a situation where she and her friend were the only ones not taking drugs, and that she thinks that many adolescents “can’t say no.” This account is in line with the study of Moilanen et al. [36], who found that adolescents were pressured to make health choices that were similar to their peers and typically followed their peers when it came to drinking or smoking. We thus argue that our participants’ wish for more education about these topics may reflect a wish to take the (peer) pressure off. Crucially, our participants generally did not talk about bad health choices they made themselves, but often mentioned others doing it.

Lastly, as discussed above, adults were an important social resource for our participants. However, some participants’ expectations that adults would simply take care of things if necessary could actually prevent the activation of this resource. This is illustrated by the following statement from Laura (f, track B), who expected that adults, without being asked for help, would automatically solve adolescents’ problems: “Perhaps, as I said earlier, they should look more at this evaluation sheet and really take action. Because I witnessed how some people really got the worst result [in the questionnaire] and from what I heard, nobody did anything about it.”

## Discussion

In this qualitative study with adolescents in the Canton of Zug, Switzerland, we found that participants often activated their resources, most prominently those available in their social networks, to develop and practice health literacy. Although context is generally considered an important determinant of (adolescent) health literacy, it often remains ambiguously defined. Our analytical focus allowed us to better understand how contextual factors, such as the quality of *relationships* (*rapport*, *trust and familiarity*, *fear of judgment*), *access* to and *participation* in the *health system*, and *pre-existing health literacy* in the form of *knowledge* and *motivations and attitudes* that adolescents brought to the table, were important *activators* and *deactivators* for adolescents’ health literacy development and practices in their daily lives. While the (de)activators we analyzed in our data are certainly not an exhaustive description of what actually constitutes context in youth health literacy development and practices, we argue that they shed light on areas for actionable recommendations. What is more, our findings may serve as a starting point for further investigations of the context which influences health literacy development and practices of adolescents. As illustrated by the many referrals between different sections of our results, it is important to note that there is a lot of overlap and interconnectedness between what we identified as (de)activators, particularly within the same category.

### Relationships

We found that simply knowing people or having access to social resources is not enough; relationships require certain qualities for adolescents to activate them as resources to develop and practice health literacy. Participants expressed a good *rapport*, *trust and familiarity*, as well as *fear of judgment* as key factors moderating the activation of their social networks. The importance of trust when dealing with health topics aligns with previous findings. For example, in an ethnographic exploration of health literacy in urban neighborhoods, Samerski [20] found that for interviewees, the primary source of advice in health matters was a trusted person. Trust and the fear of judgment are particularly relevant in adolescence: Uncertainty about others’ trustworthiness increases during adolescence [37]. Mulfinger et al. [38, p. 296], in reflecting upon the work of Moses [39], argue that “in general, adolescents – more so than adults – have to deal with age-related concerns, peer acceptance, and search for identity, which makes them a vulnerable group.” Trust in healthcare professionals was often linked to confidentiality, which is crucial in patient-provider relationships, especially for adolescents [30]. Of course, which of the factors – *trust and familiarity*, *fear of judgment*, *rapport* – came into play depended on the type of relationship at hand, and not every relationship in an adolescent’s life needed to cover all bases. Participants discussed having a variety of social resources at their disposal and knowing when to activate them, which is consistent with Mulfinger et al. [38] in their study on adolescents with mental illness.

### Health system

In line with the increasing recognition in the literature that health literacy of the individual is highly dependent on the broader system, i.e. health workers, services, organizations and policy-makers [2], we found that system-level factors were important (de)activators of adolescents’ resources. *Access* to resources and specifically having enough time to discuss and learn about health topics at school and with health providers functioned as an important activator. Importantly, access to healthcare is also related to the issue of (in)equality. Most OECD countries emphasize the importance of equal access to healthcare [40] and much health literacy literature mentions or is based on disadvantaged youth specifically [5, 6, 41, 42]. It is important to note here that our findings do not necessarily relate to this topic, as study participants were selected from an affluent canton in Switzerland, and we did not collect data on their socio-economic background. Another factor that played a surprisingly small role in our interviews were social media and digital technologies in general, despite their growing importance for health-related information, health communication and access to healthcare services [2, 43]. We attribute this finding to the rather young age of study participants and the focus of our interview guide. Regarding *participation*, Brabers et al. [44] argue that higher health literacy correlates to higher participation rates. We go a step further and suggest that this functions in a ‘virtuous circle’: When participation is facilitated by the system and health practitioners within it, it can be a powerful activator of resources. However, abilities for participation and shared decision-making need to be learned [4], which also means adapting processes to the child’s or adolescent’s development and age [45].

### Pre-existing health literacy

One important set of factors moderating the activation of resources of our participants to develop and practice health literacy was – interestingly enough – their *pre-existing health literacy*. As with *participation*, we argue that *pre-existing health literacy* acts in a ‘virtuous circle’ to develop more health literacy. As Wharf-Higgins et al. [5, p. 354] put it: “The individual accumulates knowledge through the use of health literacy skills, and in turn, their knowledge influences their health literacy. The student’s attitude towards health also influences and is influenced by his/her health literacy.” Even though adolescents are dependent on their social network and particularly adults to a certain extent, it is important to acknowledge them as “health literacy agents” [4, p. 581] who actively develop and practice health literacy. Our data nicely demonstrate both adolescents’ dependency on their social network as well as their agency in terms of pre-existing health literacy to build upon, contributing to a resources- rather than a knowledge-deficit-perspective.

### Implications

In general, awareness of how adolescents activate their resources to develop and practice health literacy is important for any professional aiming to improve adolescents’ health and wellbeing. While we show that adolescents are health literacy agents, we argue that health professionals, teachers and public health specialists as well as organizations and systems are relevant in supporting adolescents in this process, by building good relationships and trust, providing access to resources, and increasing participation opportunities and abilities.

Study participants already effectively utilized a variety of resources. Thus, increasing awareness of these non-school resources among adolescents through school health services would be worthwhile. School (health) programs and curricula also have the potential to teach children and adolescents how to participate in shared decision-making when it comes to their health and well-being, and thus how to improve activation of their resources. Moreover, addressing intimate or uncomfortable topics such as mental health, sexual health and drug use at school could contribute to reducing adolescents’ fear of judgment and thus of disclosing this information, as could explicit mention of these topics by health professionals [30]. This would lead to a shift towards more activation of resources and reduction of deactivators. Importantly, the way information is presented is often more important than the information itself [5]. We argue that students can be facilitated to talk about health topics through *other talk*, by which we mean talking about other people (in the form of vignettes, for example). We observed that participants in the interviews often talked about the health (behaviors) of others, e.g. when it came to substance use, but rarely their own. Discussing health behaviors and how to deal with them according to fictional examples relating to their lifeworld is a way to engage adolescents without them needing to disclose personal information. This is a method which is already employed in the preparation lessons as part of the school health service in Zug.

Lastly, we found that the quality of relationships adolescents had with their social network was crucial for its activation. Therefore, it could be worthwhile to reflect on social networks and their importance for adolescents also in regular school settings or in the preparation lessons. Moreover, the implications outlined above may indirectly lead to improved relationships but ultimately, we need to acknowledge that good relationships cannot be manufactured. Nevertheless, we propose that awareness of how adolescents activate their resources and the important role of good relationships is crucial.

## Limitations

Our study has certain limitations. Most importantly, our analysis was based on secondary use of data originally collected for a different purpose, i.e., the evaluation of the school health service in the canton of Zug. Thus, we did not include questions specifically targeting participants’ development and practices of health literacy, or even more specifically, about the activation of their social network, in the interview guide. Instead, the analytical focus was born out of a desire to further understand this phenomenon which emerged during initial data analysis. This also meant that we did not proactively sample for known determinants of health literacy, e.g. gender, socio-economic background or educational track [46]. In fact, there was an overrepresentation of female students and those of educational track B in our sample. Future research should avoid these shortcomings and investigate how the above-mentioned factors relate to the (de)activation of resources within adolescents’ social networks. Also related to the data collection, our sample was relatively small and consisted of students who participated voluntarily. Therefore, their health literacy levels may be higher compared to those of their peers. Finally, our analysis is based solely on self-reported data from students and relates to their experiences and perspectives. Accordingly, our results are mostly individual- and relationship-centered rather than focused on the environment, i.e., systemic or structural factors. Related to this, we did not evaluate or analyze the health literacy of other people such as teachers, parents etc. and made no observations related to organizational [47] or systemic [48] health literacy. Nevertheless, we are convinced that these first insights on the development and practices of health literacy in relation to a school health service program are of great relevance for further research in the area of adolescents’ health literacy and organizational health literacy in the school setting. Even more so as this is still in its infancy in Switzerland.

## Conclusions

In conclusion, we found that the quality of *relationships* surrounding issues of *trust*, *familiarity*, and *fear of judgment*, played a major role for adolescents to effectively activate their resources to develop and practice health literacy. *Access*, including sufficient time, was a requirement for resource activation, and opportunity for *participation* in healthcare and health information was key. Finally, participants’ *pre-existing health literacy* acted as a ‘virtuous circle’ to enable participants to activate their resources to develop higher levels of health literacy and apply it in their everyday lives, recognizing adolescents as health literacy agents.

With this study, we add to the growing body of literature demonstrating the importance of social networks, relationships and the school setting for adolescents’ health literacy, while providing some more clarity to the often ambiguously described concept of *context*, in that we highlight under which conditions participants were able to activate their resources. As we have shown, different problems and situations require the activation of different, complementary resources (e.g. different members of social networks) and are affected differently by our so-called *activators* and *deactivators*. These (de)activators not only provide an opportunity for the development of specific actions to support the development and practice of adolescents’ health literacy but could also enable future research regarding the importance of context’s potential for actionable change as adolescents learn to become active agents in their health decisions.

## Electronic supplementary material

Below is the link to the electronic supplementary material.

**Supplementary Material 1**: **Additional file 1**: Interview Guide for Manuscript Submission ID 93075789-d0a5-43a8-897f-8269dc1a3a9e.

## Data Availability

Pseudonymized data from this study is available on reasonable request from the corresponding author and with the permission of the local ethics committee (“Ethikkommission Nordwest- und Zentralschweiz”).
