# Supplementary material for: Context counts: a qualitative study on how adolescents activate social resources to develop and practice health literacy
Source: BMC Public Health. 2024 Dec 24;24:3583. doi: 10.1186/s12889-024-21138-9 (PMC11669218; doi:10.1186/s12889-024-21138-9)
Supplement: Supplementary file 1 — Supplementary Material 1: Additional file 1: Interview Guide for Manuscript Submission ID 93075789-d0a5-43a8-897f-8269dc1a3a9e. [file 12889_2024_21138_MOESM1_ESM.pdf]

Developed by Yael Rachamin
Translated by Rebekah A. Hoeks September 2024

Additional file 1

Interview Guide for Manuscript Submission ID 93075789-d0a5-43a8-897f-8269dc1a3a9e

1. **Beginning**
   a. Introduce myself
   b. Information about the interview:
   c. Time frame 20 minutes (can be broken off)
   d. Aim: To find out whether the preparation for the school doctor's visit was useful and what could perhaps be improved
   e. Ensure confidentiality (anonymous answers)

2. **Introduction: personal data**
   a. Name
   b. Age
   c. Class level, teacher

3. **Preparation lesson (PL) and coloured questionnaire**

Main question: 'Can you tell me a bit about the preparation lesson (PL)? What do you remember, what did you think of it?'

Prompts

a. Possible answers: annoying, disruptive, not important etc. What other (perhaps more important) things could have been done in this lesson?
b. How did you imagine the school doctor examination would go before the PL? What did the PL change about this expectation?
c. Do you remember the coloured questionnaire? What did you think of the questionnaire? What did you think of the questions? What was it like for you to fill in the questionnaire? What did you learn?

4. **School doctor (SD) visit**

Main question: 'Can you tell me something about how the visit to the school doctor (SD) went?'
→ Was the SD a woman or a man?
→ Were you alone during the SD visit/in groups
→ 'How was the SD visit for you?'

Prompts

a. Did you talk about health issues and problems? What type of conversation was it?
b. How long did you talk for? Would you have liked to talk longer? About what?
c. How did you feel about the SD asking you such questions? / Did it bother you that they didn't ask you anything?
d. Do you think there are many students who would talk to the SD about their problems?

e. What could be improved so that students could/would talk about health issues or problems with more ease?
f. Would you rather talk to a female/male SD about certain topics? Why yes, why no?
g. What is your understanding of ‘confidentiality’? Do you think that the SD will tell your parents or teachers what you told them? → ensure patient/doctor confidentiality if interviewee is unsure.
h. What bothered you during the visit?
i. What would you have liked to happen? What should have been different?
j. What did you particularly like?
k. What was the reason for you to take part in this interview?

5. Health topics and health in general

Main question: ‘Do you think there needs to be more information about health/health promotion for young people?
→ If yes, about what? If no, why?

Prompts

a. What do you think people should learn more about in school concerning health issues?
b. How could knowledge about health be increased?
c. What do you do when you have questions about health issues? Is there someone you can ask about health problems? Who?
d. School social work or school psychologist: Do you know them? What is your impression of them? What is the difference between them and the SD? Do the other pupils notice when you go there?
e. Are there any issues that are particularly problematic in your class or school? If yes, which ones?
f. Was/is corona an issue at school? If yes, in what way?

6. Ending

   a. Do you have any questions for me?
   b. Is there anything you would like to add, which we didn’t talk about during the interview?
   c. Thank you and goodbye, end audio recording.
